# Health risk assessment of heavy metals in marine fish to the population in Zhejiang, China

**DOI:** 10.1038/s41598-021-90665-x

**Published:** 2021-05-26

**Authors:** Jian-Long Han, Xiao-Dong Pan, Qing Chen, Bai-Fen Huang

**Affiliations:** Zhejiang Provincial Center for Disease Control and Prevention, Room No. 401, Bin-Sheng Road No. 3399, Binjiang District, Hangzhou City, 310051 China

**Keywords:** Public health, Environmental chemistry

## Abstract

Environmental pollution with toxic metals can lead to the possible contamination of the marine fish. We investigated the levels of As, Cd, Cr, Hg and Pb in 652 marine fish samples (15 species) collected from coastal areas of Zhejiang, China and estimated their health risk. Mean concentrations of As, Cd, Cr, Hg and Pb were 0.783, 0.009, 0.114, 0.031, 0.043 mg/kg wet weight. The average estimated daily intakes (EDIs) for As, Cd, Cr, Hg and Pb were 1.214, 0.014, 0.177, 0.048 and 0.067 μg/kg bw/day. The risk assessment at mean exposure level showed that there was no health risk associated with these elements through consumption of marine fish. However, potential health risk may exist for high exposure consumers considering the possible contamination of As and Hg. Given that the different levels of certain elements in marine fish in China, this study provides a scientific basis for food safety assessment and suggestions for risk management.

## Introduction

Fish is of great interest for consumers considering its high quality protein, polyunsaturated fatty acids, vitamin-B, and other nutrients. However, environmental pollution becomes more and more serious in some coastal and estuarine areas with rapid industry development. Marine organisms can accumulate these contaminants in their muscle tissues and give rise to potential health risk.

Heavy metals have been considered a serious global environmental threat^1^. Marine fish can absorb toxic metals from the surrounding water and sediment as well as through their food^2–5^. For example, Tarley et al.^6^ observed the Pb with the mean level of 2.15 mg/kg in canned sardines from Brazil. Ogundiran et al.^7^ found the Cd with mean level of 0.19 mg/kg in European pilchard from south western Nigeria. Marcotrigiano et al.^8^ reported a high average level of Hg (6.53 mg/kg) in spiny dogfish from Italy. Leung et al.^9^ observed level of Pb (8.62 mg/kg) in tilapia from Pearl River Delta of China. All these studies indicated that possible contamination of marine fish by heavy metals.

The high exposure of heavy metals and metalloid elements has the confirmative negative effects to human health. Cd, commonly presented as inorganic compounds in the 2^+^ oxidation state, can cross various biological membranes and lead to neurological disorders, carcinogenic effects and skeletal weakness and defects^10^. Chronic Hg exposure impacts the pituitary gland and the liver and leads to a compromise of the immune system^11^. Pb has been proved to be associated with neurological problems, haematological effects, renal failure, hypertension and cancer^12^. It is therefore reasonable to hypothesize that consumption of marine fish contaminated by heavy metals has the potential health risk.

Zhejiang province, a coastal area of East China Sea, is a rapidly developing region with a high population density, where heavy metal is one of the most important environmental issues^13^. Previous studies have revealed the heavy metal pollution in the soil and nearshore sediments from Zhejiang^14–16^. However, to our knowledge, few studies on the level of heavy metals in marine fish and exposure assessment in Zhejiang have been reported^17^.

The main aims of this study were to analyze heavy metals in marine fish from Zhejiang province and evaluate the health risk based on daily consumption of local residents. The results of our study may provide some insight into heavy metal accumulation in marine fish and serve as a basis for comparison to other regions both in China and worldwide.

## Materials and methods

### Sampling and sample preparation

**Figure 1:**
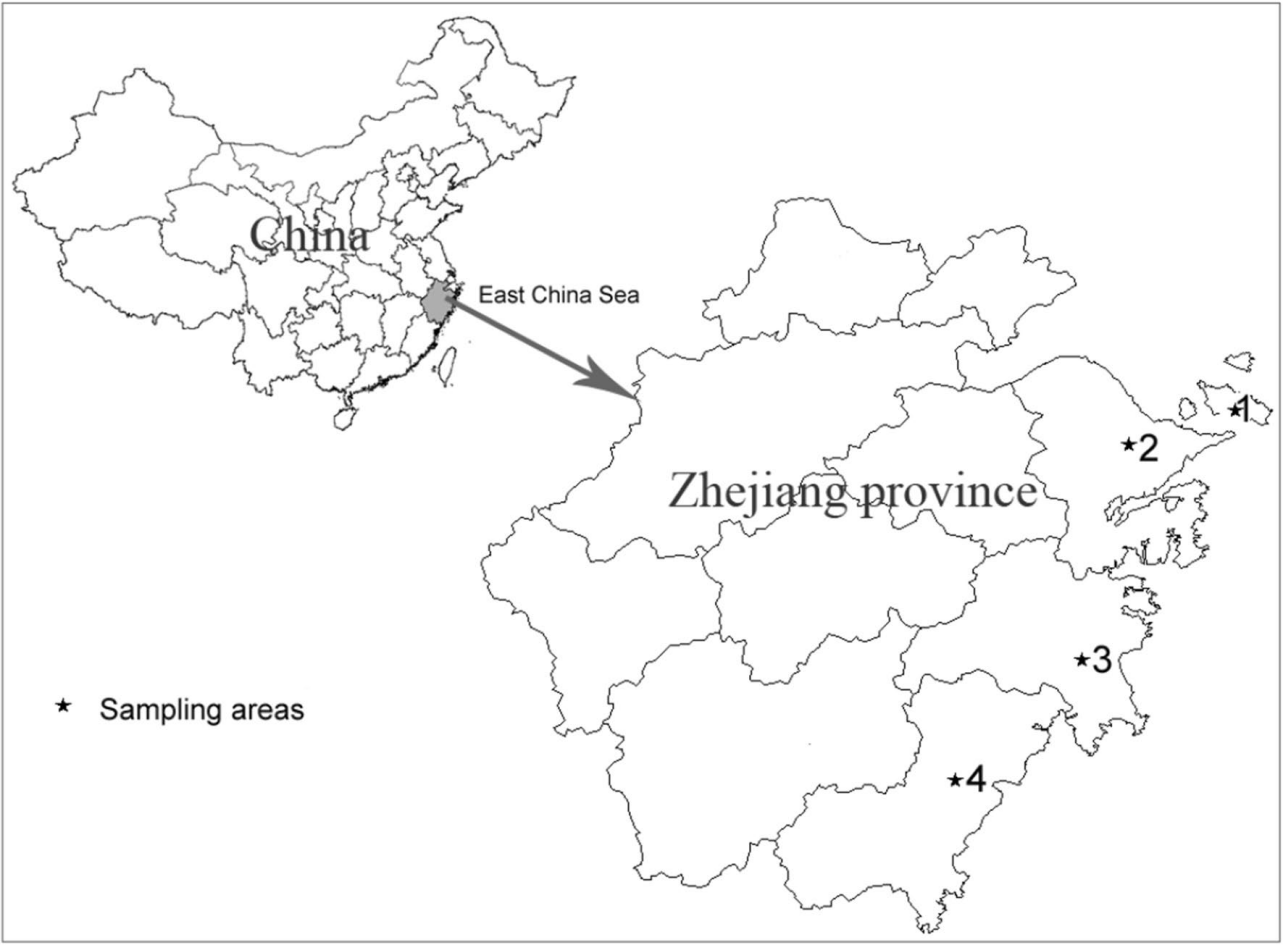
Simple map of the sampling areas of Zhejiang province, China.

The simple map of sampling place (Zhejiang) was shown in Fig. 1 which was drawn by software of MapGIS K9 SP2 free trial edition (Zondy Cyber Comp., China, http://www.mapgis.com/index.php/index-view-aid-280.html). Total 652 marine fish samples (15 species) were collected at local markets from 2019 to 2020. Four coastal cities marked by asterisks in Fig. 1 were selected as the sampling areas named by 1, 2, 3, and 4. All samples were collected and refrigerated at − 20 °C until later analysis in the laboratory. The storage period was no more than 7 days.

### Chemical analysis

The concentrations of As, Cd, Cr, Cu, Hg, Ni, and Pb were tested according to the previous report^18–20^. Briefly, samples (0.5–1.0 g) were digested in acid-clean Teflon vessels containing 6 mL HNO3 in a Mars-6 microwave digestion system (CEM, Charlotte, NC, USA). The samples in closed vessels were heated at 190 °C for 20 min. After digestion, the residue was heated at 150 °C till nearly dry. Then, it was diluted to 20 mL by ionized water for instrumental analysis. As, Cd, Cr, Cu, Hg, Ni, and Pb in all samples were tested using NexION 300 ICP-MS (Perkin Elmer, Inc., Shelton, CT USA). For quality assurance and quality control purposes, sample blanks, certified reference materials (CRMs), and duplicates of the samples (10% of the load) were applied in each batch of treated samples. The data used for exposure estimates were according to the recommendation of the report Reliable Evaluation of Low-Level Contaminations of Food issued by WHO^21^. Thus, a value of ^1^/_2_ LOD was assigned to all results below the LOD, where the proportion of < LOD results is not > 60%.

### Analysis of CRMs

The accuracy of the analytical procedures was verified by analysis of appropriate certificated reference materials (CRMs) using the same digestion and analytical methods. Two CRMs (Table 1) were purchased from National Research Center for Certified Reference Materials, China (NRCCRM). Quantitative results (within 10% of the certified value) were obtained for each metal in each CRM. Limits of Detection (LODs) were defined as three times the standard deviation of ten runs of blank measurements. LODs of As, Cd, Cr, Hg and Pb were 0.003, 0.001, 0.005, 0.0003 and 0.004 mg/kg respectively.

**Table 1:** Determination of certified reference materials.

	GBW10050 shrimp		GBW08573 yellow croker	
	Certified (mg/kg)	Measured (mg/kg)	Certified (mg/kg)	Measured (mg/kg)
As	2.5	2.4 ± 0.4	5.08 ± 0.39	4.82 ± 0.66
Cd	0.039 ± 0.002	0.035 ± 0.005	0.015	0.011 ± 0.007
Hg	0.049 ± 0.008	0.051 ± 0.004	0.169 ± 0.018	0.155 ± 0.017
Pb	0.2 ± 0.05	0.17 ± 0.07	0.25	0.22 ± 0.09
Cr	0.35 ± 0.11	0.32 ± 0.15	0.43	0.41 ± 0.05

### Fish consumption data

The fish consumption data used in this report was extracted from the Food Consumption Survey conducted in Zhejiang province, China in 2008 by the Zhejiang Food and Drug Administration based on our previous reports^22–24^. The representative sample of participants included 9798 people, who were questioned twice about their last 24-h consumption. The selection of interviewed people and the moment of the interview were chosen in order to obtain a representative consumption profile of the population over 1 year. The estimated fish intake of adult was 108.50 g/day per person.

### Exposure estimates

The targeted hazard quotient (THQ) and hazard index (HI) were used to estimate health risk according to US EPA’s IRIS database^25^. We used the mean and 97.5th percentile of obtained elements concentration to represent the consumers with average and high exposure, respectively^26^. The sum of all THQs for each element is referred to as the HI. The formulas are as follows:

1 $${\text{Exposure}}\;{\text{dose}} = \frac{Ci \times Di \times Ed}{{Bw \times At}}$$

2 $${\text{Tageted}}\;{\text{hazard}}\;{\text{quotient}}\left( {{\text{THQ}}} \right) = \frac{{{\text{exposure}}\;{\text{dose}}}}{RfD}$$

3 $${\text{Hazard}}\;{\text{index}}\left( {{\text{HI}}} \right) = \sum\limits_{k = 1}^{n = k} {{\text{targeted}}\;{\text{hazard}}\;{\text{quotient}}}$$

Ci is the average or P97.5 concentration of the element in the fish (mg/kg); Di is the daily intake of fish (108.5 g/capita/day); Ed is the average exposure duration (e.g., 70 years); Bw is the average weight (60 kg for adults and 30 kg for children); At is the average lifetime (e.g., 70 years). RfD is the oral reference dose (μg/kg/day, 3 for As, 0.8 for Cd, 3000 for Cr, 0.14 for Hg, 1.5 for Pb); According to US EPA guidelines for assessing conservative risk, HI were calculated by sum of the THQ. When HI < 1, no health risk is expected to occur; If HI ≥ 1, there is moderate or high risk for adverse human effects.

### Statistical analysis

The statistical analysis was performed by software of Statistical Analysis System (SAS V9.42) (SAS Institute Inc., Cary, NC, USA). Normality and equality of variances of the data were analyzed using Kolmogorov–Smirnov and Levene tests, respectively. Collected data were not normally distributed, and data were transformed into log. One-way analysis of variance (ANOVA) was used to assess whether heavy metals in marine fish and health index (HI) for adults and children varied significantly (*P* < 0.05) in different sampling areas.

## Results and discussion

### Heavy metals in marine fishes

**Figure 2:**
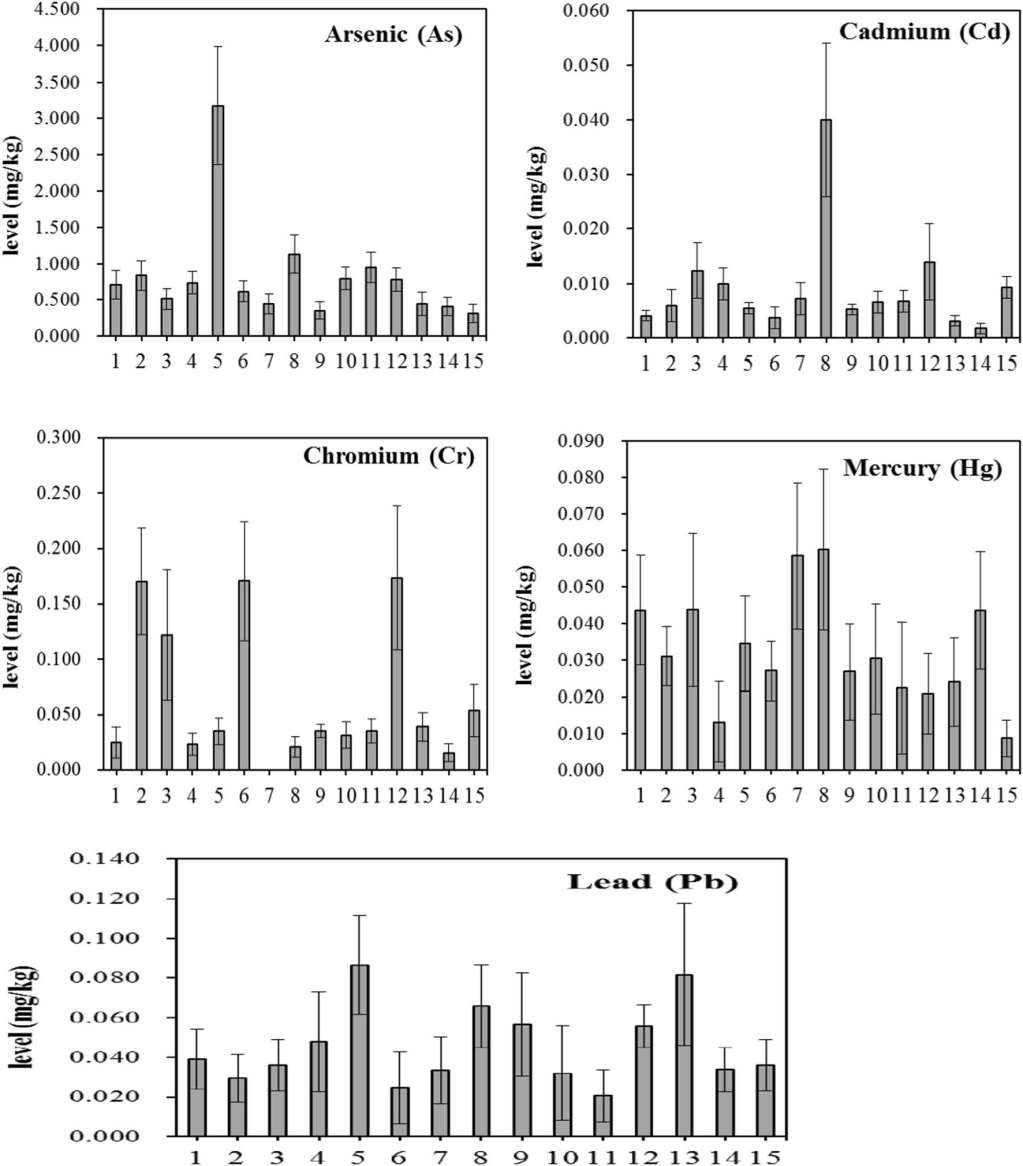
The distribution of the As, Cd, Cr, Hg and Pb in marine fish from Zhejiang province, China (mean ± standard error). 1, Large yellow croaker (*Larimichthys crocea*); 2, Little Yellow Croaker (*Larimichthys polyactis*); 3, Largehead hairtail (*Trichiurus lepturus*); 4, Light maigre (*Collichthys lucidus*); 5, Flounder (*Pleuronectiformes*); 6, Sea bass (*Lateolabrax japonicus*); 7, *Leuciscus waleckii*; 8, Pacific saury (*Cololabis saira*); 9, *Miichthys miiuy*; 10, *Pneumatophorus japonicus*; 11, *Scomberomorus niphonius*; 12, Pomfret (*Pampus argenteus*); 13, Flathead grey mullet (*Mugil cephalus*); 14, Bennett (*Ilisha elongata*); 15, *Harpadon nehereus.*

Of all fish samples we measured, the highest concentrations of As, Cd, Cr, Hg and Pb were found to be 17, 0.76, 0.836, 0.289 and 1.48 mg/kg wet weight, respectively. The mean levels, P97.5 and the range (average levels in different species) were listed in Table 2. The levels of analyzed elements in 15 fish species were presented in Fig. 2. The comparison of these elements in fish with some previous studies was showed in Table 3. The extent of potential pollution in fish can be evaluated by comparing with the maximum allowable concentrations (MAC) recommended by Chinese legislation^27^.

**Table 2:** The concentration of heavy metals in marine fish from Zhejiang province (mg/kg).

Elements	n	Mean ± SD^a^	P97.5^a^	Average range	MAC^b^	No. of > MAC
As	643	0.783	3.445	0.315–3.172	–	–
Cd	652	0.009	0.046	0.002–0.04	0.1	1
Cr	189	0.114	0.391	0.016–0.173	2	0
Hg	650	0.031	0.138	0.009–0.060	–	–
Pb	633	0.043	0.261	0.021–0.082	0.5	3

^a^Target analytes with concentrations lower than LOD were treated as one-half of LOD when calculating the mean values; SD, standard deviation.

^b^Maximum allowable concentrations of contaminants in foods.

**Table 3:** Comparison of the levels of toxic elements in fish and exposure estimates to some previous studies.

	Area	N	Mean level (mg/kg)	Exposure (μg/kg bw/day)	References
As	Bangladesh (Dhaka)	–	0.4–1.3	–	Rahman et al. (2012)^29^
	Patuakhali	–	0.3	–	Islam et al. (2015)^39^
	Croatia	61	0.56–23.3	0.2–12(iAs)	Juresa et al. (2010)^28^
	China (Zhejiang)	30	0.325–1.244	0.6–2.3	Liu et al. (2020)^17^
	South Korea	–	1.23–44.54	–	Islam et al. (2010)^30^
	China (Pearl River Delta)	711	0.003–1.53	–	Leung et al. (2014)^9^
	China (Zhejiang)	643	0.783 (0.315–3.175)	1.416	This study
Cd	Oman	873	0.0049–0.036	–	Al-Busaidi et al. (2011)^35^
	Croatia	61	0.002–0.142	0.1–0.2	Juresa et al. (2010)^28^
	China (South China Sea)	255	0.006–0.050	–	Gu et al. (2017)^34^
	China (Zhejiang)	30	0.004–0.007	0.008–0.013	Liu et al. (2020)^17^
	South Korea	–	LOD-0.13	–	Islam et al. (2010)^30^
	China (Pearl River Delta)	711	0.02–0.06	–	Leung et al. (2014)^9^
	China (Zhejiang)	652	0.009 (0.002–0.040)	0.016	This study
Hg	Oman	873	0.015–0.101	–	Al-Busaidi et al. (2011)^35^
	Croatia	61	0.134–0.373	0.2–1	Juresa et al. (2010)^28^
	China (Zhejiang)	30	0.002–0.008	0.005–0.015	Liu et al. (2020)^17^
	South Korea	–	0.01–0.24	–	Islam et al. (2010)^30^
	China (Zhejiang)	650	0.031 (0.009–0.060)	0.056	This study
Pb	Bangladesh (Dhaka)	–	0.4–2.1	–	Rahman et al. (2012)^35^
	Patuakhali	–	0.7	–	Islam et al. (2015)^39^
	Oman	873	0.029–0.196	–	Al-Busaidi et al. (2011)^35^
	Croatia	61	0.007–0.150	0.1–0.4	Juresa et al. (2010)^28^
	Italy	41	0.008	0.003	Malavolti et al. (2020)^40^
	China (South China Sea)	255	0.13–0.68	–	Gu et al. (2017)^34^
	China (Zhejiang)	30	0.004–0.014	0.008–0.038	Liu et al. (2020)^17^
	South Korea	–	0.05–0.74	–	Islam et al. (2010)^30^
	China (Pearl River Delta)	711	0.03–8.62	–	Leung et al. (2014)^9^
	China (Zhejiang)	633	0.043 (0.021–0.082)	0.078	This study
Cr	Bangladesh (Dhaka)	–	0.09–0.4	–	Rahman et al. (2012)^29^
	Patuakhali	–	0.7	–	Islam et al. (2015)^39^
	China (South China Sea)	255	0.18–0.85	–	Gu et al. (2017)^34^
	South Korea	–	0.09–1.32	–	Islam et al. (2010)^30^
	China (Pearl River Delta)	711	0.2–0.65	–	Leung et al. (2014)^9^
	China (Zhejiang)	189	0.114 (0.016–0.173)	0.206	This study

#### Arsenic (As)

Total arsenic concentration in fish varied over a range of 0.005–17 mg/kg with a mean of 0.783 mg/kg. The result was similar with the study of Liu et al.^17^, who observed 0.325–1.244 mg/kg in fish samples from East China Sea. Previous reports also found 0.56–23.3 mg/kg in fish from Croatia^28^, 0.4–1.3 mg/kg in fish from Bangladesh (Dhaka)^29^, 1.23–44.54 in fish from South Korea^30^. Our results showed that the highest average level of As (3.172 mg/kg) was found in flounder (*Pleuronectiformes*).

Arsenic can be presented in several organic (trivalent and pentavalent arsenic) and inorganic (elemental, trivalent and pentavalent arsenic) forms. Organic As showed low toxicity does not accumulate due to the rapid excretion in the human body^31^. Up to 90% of As in fish muscle is present in the non-toxic arsenobetain form^32,33^. However, according to MAC (0.1 mg/kg) for inorganic As (iAs) in China, there could be some samples exceeding the MAC in this study.

#### Cadmium (Cd)

The concentration of Cd was found at the mean of 0.009 mg/kg (average range of 0.002–0.040 mg/kg). One sample (pomfret, *Pampus argenteus*) of 652 contained higher level than the MAC of 0.1 mg/kg. Previous report also found the average level of 0.004–0.007 mg/kg in fish samples from East China Sea^17^, 0.02–0.06 mg/kg in fish samples from Pearl River Delta of China^9^, 0.006–0.050 mg/kg in fish from South China Sea^34^. We also found that Pacific saury (*Cololabis saira*) contained the highest level of Cd (0.040 mg/kg) among the 15 fish species.

#### Mercury (Hg)

The Hg level was observed with a mean of 0.031 mg/kg (average range of 0.009–0.060 mg/kg). Our results were lower than those reported in other areas of Croatia, Oman and South Korea, where the levels were ranged from 0.015 to 0.373 mg/kg^28,30,35^. Two fish species of Pacific Saury (*Cololabis saira*) and *Leuciscus waleckii* showed higher concentrations of 0.060 mg/kg and 0.058 mg/kg than other fishes.

Mercury is present in the environment in organic and inorganic chemical forms, each showing different characteristics including mobility and toxicity. Among these chemical forms, methylmercury (MeHg) is well-known as a serious toxicant to human body. The nervous system is the primary target organ for MeHg poisoning and the brain of developing fetus is more sensitive than that of the adults^36^ MeHg generally accounts for 75–100% of the total Hg present in most fish species^37^. Furthermore, single species especially in predatory fish with older or larger body may have higher levels of accumulated Hg^38^. Although the level of Hg found in this study was low, concerns still need to be made in terms of the potential pollution.

#### Lead (Pb)

The mean level of Pb was 0.043 mg/kg (average range of 0.021–0.082 mg/kg). According to the current MAC of 0.5 mg/kg, 99.5% of total samples were acceptable on Pb contamination level. As shown in Table 3, the reports on Pb levels in fish from China varied with a great extent^9,17,34^. Similar levels with our data were reported by Liu et al.^17^. In some polluted area, such as Patuakhali, mean Pb level was as high as 0.7 mg/kg^39^. Low level of Pb (0.008 mg/kg) in fish from Italy was observed^40^.

Lead is one of the main pollutants in the environment and naturally occurs in rocks, soils and in the hydrosphere. Once discharged into the marine environment, Pb is easily absorbed by fish and accumulated in the body tissues, bones, gills, kidneys, liver and scales^41^. Our results showed that different fishes accumulated diverse lead concentrations.

#### Chromium (Cr)

Average concentration of Cr in analyzed fish was 0.114 mg/kg (average range of 0.016–0.173 mg/kg). High mean levels for Cr were found in pomfret (*Pampus argenteus*) with 0.173 mg/kg, sea bass (*Lateolabrax japonicas*) with 0.71 mg/kg and little yellow croaker (*Larimichthys polyactis*) with 0.17 mg/kg. Our results were lower than those found in other place of China which showed 0.18–0.85 mg/kg in South China Sea and 0.2–0.65 mg/kg in Pearl River Delta^9,34^. In South Korea, the Cr concentrations in fish were 0.08–1.32 mg/kg^30^.

### Comparison of heavy metals in different sampling areas

As shown in Table 4, levels of As, Cd, Cr, Hg and Pb in marine fish from four areas (1, 2, 3 and 4) were compared. For As, Hg and Pb mean levels in area 1 were significantly lower than the other sampling areas (*P* < 0.05). Cr levels in area 1 and 2 were obviously lower than area 3 and 4 (*P* < 0.05). There were no statistical difference for Cd levels in four sampling areas (*P* > 0.05). It indicates that the heavy metals contaminated in marine fish may derive from different sources.

**Table 4:** Comparison of different heavy metals in marine fish from four sample areas in Zhejiang, China.

Sampling area	Median (P25, P75) (mg/kg wet weight)				
	As,	Cd	Cr	Hg	Pb
1	0.628 (0.414, 0.699)^a^	0.008 (0.002, 0.009)	0.112 (0.075, 0.177)^a^	0.017 (0.006, 0.022)^a^	0.019 (0.007, 0.032)^a^
2	0.798 (0.352, 0.827)^b^	0.016 (0.003, 0.021)	0.109 (0.069, 0.180)^a^	0.034 (0.018, 0.041)^b^	0.062 (0.029, 0.072)^b^
3	0.830 (0.413,0.941)^b^	0.011 (0.002, 0.023)	0.180 (0.105,0.251)^b^	0.036 (0.017, 0.049)^b^	0.055 (0.031, 0.076)^b^
4	0.867 (0.379, 0.957)^b^	0.009 (0.003, 0.018)	0.190 (0.113, 0.264)^b^	0.057 (0.021, 0.068)^b^	0.059 (0.028, 0.071)^b^

Means followed by different letters (a and b) in each column are significantly different (*P* < 0.05).

### Estimated daily intake (EDI) of heavy metals

The EDIs of adults were showed in Table 5. The 97.5th percentile (P97.5) level was used to represent the high exposed consumers of the distribution. The mean intakes of As, Cd, Cr, Hg and Pb through marine fish were estimated to be 1.214, 0.014, 0.177, 0.048, and 0.037 μg/kg bw/day. The P97.5 daily intakes of As, Cd, Hg and Pb were 5.34, 0.071, 0.606, 0.214, and 0.405 μg/kg bw/day. Our results of mean EDIs were similar with the study of Liu et al.^17^, and lower than the report of Rahman et al.^29^. Comparing with the recommended safe value (showed in Table 5), the P97.5 daily intakes of As exceeded the safe limit. It indicates that the long-term large consumption of marine fish may have potential health risk for As in Zhejiang.

**Table 5:** Estimated exposures to As, Cd, Cr, Hg and Pb for consumption of marine fish from Zhejiang province.

	Exposure dose for adults (μg/kg bw/day)		THQ for adults		HI for adults		Exposure dose for children (μg/kg bw/day)		THQ for children		HI for children	
	Mean	P97.5	Mean	P97.5	Mean	P97.5	Mean	P97.5	Mean	P97.5	Mean	P97.5
As	1.416	6.230	0.472	2.077	0.945	4.278	2.832	12.459	0.944	4.153	1.889	8.556
Cd	0.016	0.083	0.020	0.104			0.033	0.166	0.041	0.208		
Cr	0.206	0.707	0.000	0.000			0.412	1.414	0.000	0.000		
Hg	0.056	0.250	0.400	1.783			0.112	0.499	0.801	3.565		
Pb	0.078	0.472	0.052	0.315			0.156	0.944	0.104	0.629		

### Health risk assessment

The targeted hazard quotient (THQ) and hazard index (HI) was used for the risk assessment. The recommended reference doses (RfDs) or safe values were based on previous report^2^. As shown in Table 5, for the mean exposure, the data of THQs for both adults and children were all less than 1, which indicated that there was no potential health risk to general people. However, the THQ for As and Hg at the high exposure level (P97.5) was more than 1. HI calculated by the sum of THQ was adopted for estimating the total risk of these elements. The value of HI at mean exposure level for children was more than 1. Concerns might be paid for the high exposure of these elements by the fish consumption.

**Figure 3:**
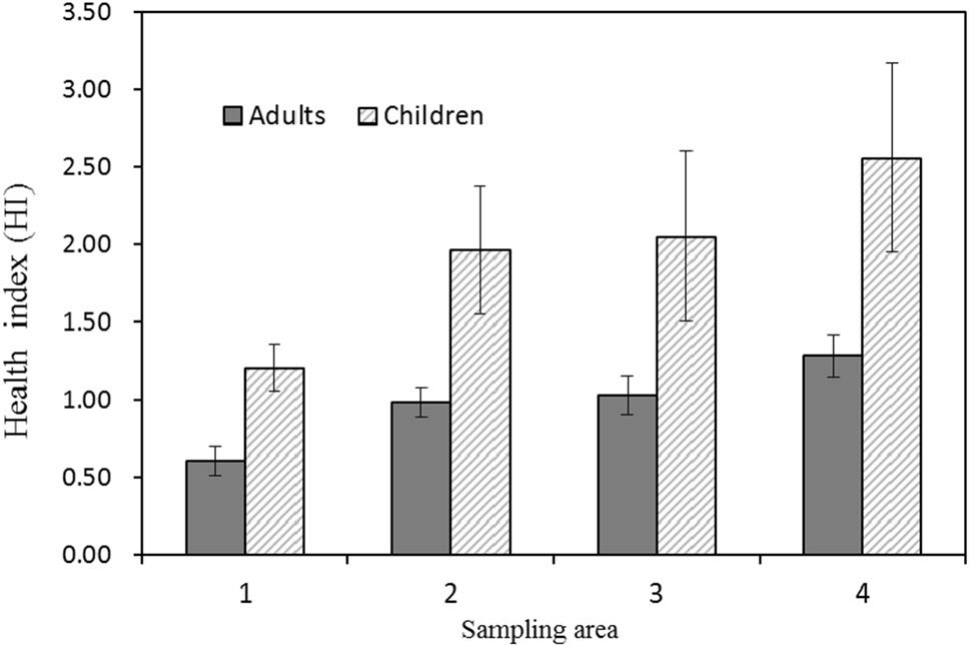
The health index (HI) of heavy metals for adults and children in different sampling areas (mean ± standard error).

The difference of HI (mean level) for adults and children in different sampling areas was investigated. As shown in Fig. 3, HIs for adults and children in sampling area 1 were obviously lower than other sampling areas (*P* < 0.05).There were no obvious difference for HIs among sampling areas 2, 3 and 4 (*P* > 0.05).

It should be noted that coastal area of China are now facing great challenges in regard to heavy metal contamination^42^. Zhejiang coastal area is typical transitional zone between land and ocean, receiving a large amount of anthropogenic pollutants^43^. Our findings are consistent with previous studies on the coast of Zhejiang, indicating that As, Pb, and Cd are the main pollutants^14^.

Furthermore, the THQ and HI widely used for evaluation of the health risk has some obvious shortages^42^: (1) Only determined targets are considered while the other potential hazardous pollutants are ignored; (2) The mutual effects of selected pollutants are ignored for calculating the THQ. (3) The toxicities of heavy metals are related with their chemical forms^44,45^. For example, methylmercury (MeHg), inorganic arsenic, Cr(VI) in aquatic organisms showed higher toxicity than their other forms. There are still some limitations in this study. For example, vulnerable populations (e.g. pregnant women) and other exposure pathway were not considered here. Therefore, the current risk assessment method for heavy metals might be improved in the further.

## Conclusion

The mean levels of As, Cd, Cr, Hg and Pb in marine fish collected from coastal area of Zhejiang (located in southeast of China) were all below their MAC of China. The risk assessment showed that mean daily exposures of these elements by marine fish consumption were lower than the reference values of JECFA, EPA or EFSA. We concluded that the consumption of marine fish from Zhejiang had low health risk to general people. However, considering the high exposure level (the P97.5 estimation), the HI of all elements was more than 1, which indicated consumers especial vulnerable populations (e.g. children, pregnant women) may experience some adverse health effects.

Accordingly, the regular monitoring of heavy metals and metalloid elements in fish is recommended in this area. We will investigate the possible sources of toxic elements in marine fish and their distribution in fish bodies in our future studies.
